# Molecular Characteristic, Antibiotic Resistance, and Detection of Highly Immunoreactive Proteins of Group B Streptococcus Strains Isolated From Urinary Tract Infections in Polish Adults

**DOI:** 10.3389/fmicb.2022.809724

**Published:** 2022-03-22

**Authors:** Anna Dobrut, Dorota Ochońska, Ewa Brzozowska, Sabina Górska, Jolanta Kaszuba-Zwoinska, Monika Gołda-Cępa, Andrzej Gamian, Monika Brzychczy-Wloch

**Affiliations:** ^1^Department of Molecular Medical Microbiology, Chair of Microbiology, Faculty of Medicine, Jagiellonian University Medical College, Kraków, Poland; ^2^Laboratory of Medical Microbiology, Hirszfeld Institute of Immunology and Experimental Therapy, Polish Academy of Sciences, Wrocław, Poland; ^3^Laboratory of Microbiome Immunobiology, Hirszfeld Institute of Immunology and Experimental Therapy, Polish Academy of Sciences, Wrocław, Poland; ^4^Chair of Pathophysiology, Faculty of Medicine, Jagiellonian University Medical College, Kraków, Poland; ^5^Faculty of Chemistry, Jagiellonian University, Kraków, Poland

**Keywords:** *Streptococcus agalactiae*, urinary tract infection, molecular characterization, antibiotic resistance, cell lines adherence, immunoreactive proteins, group B streptococcus

## Abstract

Group B streptococcus (GBS) is one of the uropathogens that causes urinary tract infections (UTIs). The aims of this article were molecular characterization, an analysis of antimicrobial susceptibility profiles, adherence to bladder endothelial cells, and the detection of immunoreactive proteins of 94 clinical strains of GBS isolated from adult Polish patients with UTI. Antibiotic susceptibilities were determined by disk diffusion. Serotyping and Alp family genes detection were studied using multiplex PCR. Genetic profiles were determined by pulsed-field gel electrophoresis. The adherence ability of the studied strains was estimated by incubation on human bladder microvascular endothelial cell line. Immunoreactive proteins were studied by immunoblotting. Antibiotic susceptibility investigation revealed that 22% of GBS strains were resistant to erythromycin, whereas 18% demonstrated resistance to clindamycin. cMLS_B_ was present in 76% of the resistant strains, M phenotype was detected in 14%, whereas iMLS_B_ was present for 10%. The most common serotype was serotype III (31%), followed by serotype V (27%), and serotype Ia (17%). The genes that dominated among other Alp genes were: *epsilon* (29%), *alp*2 (27%), and *rib* (23%). The most common co-occurring serotypes and Alp genes were: Ia and *epsilon*, III and *rib*, III and *alp*2, V and *alp*2, and V and *alp*3 (*p* < 0.001). The PFGE method showed high clonality for serotype V and cMLS_B_ (*p* < 001). The PFGE method showed high clonality for serotype V. Furthermore, this serotype was significantly associated with the cMLS_B_ phenotype (*p* < 0.001). The most common immunoreactive proteins demonstrated masses of 50 kDa and 45–47 kDa. Although examined GBS isolates showed high genetic diversity, immunoreactive proteins were common for most of the studied GBS isolates, which may indicate their conservation, and allows to consider them as potential immunodiagnostic markers. Although the examined GBS isolates showed high genetic diversity, immunoreactive proteins were shared by most of the studied GBS isolates. It may indicate their conservation, thus allowing to consider them as potential immunodiagnostic markers.

## Introduction

Urinary tract infection (UTI) is a severe health issue, which affects 150 million people each year worldwide and can be a cause of morbidity in infants, women of childbearing age, and elderly patients (Stamm and Norrby, 2001; Flores-Mireles et al., 2015; Sullivan et al., 2017). UTI may affect both lower (bladder infection, cystitis) and upper (kidney infection, pyelonephritis) urinary tract and can cause an asymptomatic bacteriuria (Lane and Takhar, 2011). Accompanying symptoms of bladder infections are dysuria, suprapubic pain, urgent and frequent urination, or hematuria, whereas pyelonephritis symptoms are characterized by cystitis with additional flank pain, costovertebral-angle tenderness, fever, nausea, or vomiting (Flores-Mireles et al., 2015). More serious outcomes include pyelonephritis with sepsis, renal damage in children, frequent recurrence, and complications affected by frequent antibiotic intake. UTI risk factors include, above all, female gender related to women’s anatomy, obesity, diabetes, a prior UTI, genetic susceptibility, and sexual activity (Flores-Mireles et al., 2015). One of the pathogens that causes UTI is *Streptococcus agalactiae* (group B streptococcus, GBS), which is responsible for 2–3% of cases. However, little is known about the GBS etiology in UTI. GBS constitutes a severe problem for pregnant women, elderly patients, and immunosuppressed individuals (Sullivan et al., 2017). Therefore, from an epidemiological point of view, it is important to conduct detailed characteristics of GBS isolates, both phenotypically and genetically.

Widespread antibiotic intake in recent decades had contributed to the growth of resistance to antibiotic therapy. Two main resistance mechanisms characteristic for GBS are encoded by *erm* (erythromycin ribosome methylase) genes, namely *erm*A and *erm*B, in which the product—methylase 23S rRNA—is responsible for the methylation of erythromycin and clindamycin receptor sites in ribosomes. The expression of these genes is described as the MLS_B_ phenotype, which is related to cross-resistance to macrolides, lincosamides, and streptogramins B. The MLS_B_ phenotype can occur as constitutive macrolide–lincosamide–streptogramin B (cMLS_B_) resistance and inductive macrolide–lincosamide–streptogramin B (iMLS_B_) resistance (Gherardi et al., 2007; Brzychczy-Wloch et al., 2010).

One of the virulence factors, on the basis of which GBS can be typed, is capsular polysaccharide (CPS), and the differences in its structure allowed to distinguish 10 serotypes: Ia, Ib, and II to IX. The diversity in serotype distribution is related not only to, particularly, infection source but also to latitude and ethnic origin (Gherardi et al., 2007; Brzychczy-Włoch et al., 2012). For example, the most common serotype in UTI is serotype III (Sullivan et al., 2017).

GBS classification can also be conducted by the characterization of the presence of the surface proteins belonging to alpha-like protein (Alp) family. Among the members of Alp family, the following proteins are listed: Alpha-C protein, Rib, Alpha-like protein 1, Alpha-like protein 2 (Alp2), Alpha-like protein 3 (Alp3), and Alpha-like protein 4 (Alp4). These are encoded by *bca*, *epsilon*, *rib*, *alp*2, *alp*3, and *alp*4 genes, respectively (Gherardi et al., 2007). This protein’s family is well known for its immunogenic character, and it also demonstrates its virulence properties thorough the ability to bind host’s epithelial cells.

The most common genotyping method is pulsed-field gel electrophoresis (PFGE) used for the separation of large DNA molecules after digesting them with a unique restriction enzyme, which is applied to distinguish specific bacterial clones among clinical isolates (Skjaervold et al., 2004; Brzychczy-Włoch et al., 2012).

In recent decades, scientists have increasingly focused on immunogenic bacterial proteins, which can be considered as components of innovative subunit vaccines and may protect from several bacterial infections. Immunogenic proteins are also described in the context of biomarkers in immunodiagnostic assays. There are a number of GBS proteins known for their immunogenic character. Except for Alp proteins mentioned above, the following proteins have been described: peptidase c5a, laminin-binding protein Lmb, fibrinogen-binding protein FsbA, immunogenic bacterial adhesin BibA, and the conservative protein Sip (Ji et al., 1997; Johri et al., 2006; Papasergi et al., 2013; Yao et al., 2019; Dos Santos et al., 2020).

Due to the lack of detailed molecular and phenotypic characterization of GBS isolated from Polish patients with UTI, and, moreover, the fact that little is known about this phenomenon worldwide, the aim of our paper was to determine the frequency of an occurrence of resistant phenotypes to macrolides in GBS isolates originating from patients with UTI. The distribution of particular serotypes and genes coding surface Alp proteins of strains representing chosen chosen macrolide resistance phenotypes, and detection of immunoreactive, conservative and specific GBS proteins was also investigated.

## Materials and Methods

### Study Population and Specimen Collection

Ninety-four GBS strains were isolated from urine samples collected from adult patients with symptoms of UTI in the course of routine diagnostic procedures in the Microbiological Diagnostic Laboratory at Jagiellonian University Medical College in Kraków. The average age of examined patients was 54 years old (the median value was 57 years old), three-fourths were women, and one-fourth constituted men. The study was approved by Jagiellonian University Bioethical Committee decision No. KBET/153/B/2014. The consent obtained from the participants was both informed and written.

Bacteriuria had been detected by cultivation of 1 μl of urine on Columbia sheep blood agar (Difco) in aerobic conditions for 24 h at 37°C. Colony forming unit (CFU)/ml ≥ 10^5^ was interpreted as significant bacteriuria.

Species identification was carried out by the isolation of colonies with GBS morphology from growth medium and tested by the CAMP test, by the API Strep kit (bioMérieux, Craponne, France), and by the latex-agglutination group kit, Slidex Strepto kit (bioMérieux, Craponne, France), for GBS identification. In doubtful cases, identification based on PCR with species-specific primers was performed (Ke et al., 2000). Bacterial strains were stored at −80°C. As a positive control, the following reference strains were used: 2134 DSM (*S. agalactiae*; serotype II), 12403 ATCC (*S. agalactiae;* serotype III), and BAA-611 ATCC (*S. agalactiae*; serotype V).

### Molecular Characteristic of Group B Streptococcus

#### Macrolide Resistance Characterization

Macrolide susceptibility testing was performed by the disk diffusion method. The distinction between cMLS_B_ and iMLS_B_ was made by the cultivation of the examined GBS strain on a growth medium with two discs (Oxoid), erythromycin (15 mg) and clindamycin (2 mg), placed on the growth medium at a distance of 12–16 mm between the edges of the disks. The phenotype was identified by interpreting resistance according to The European Committee on Antimicrobial Susceptibility Testing (2018) guidelines (EUCAST v 8.1, 2018)^1^. Resistance to both antibiotics indicated constitutive phenotype (cMLS_B_), while inductive phenotype (iMLS_B_) was identified in GBS strains resistant to erythromycin with induced resistance to clindamycin visible as a truncated zone on the side of the erythromycin disk (D-zone). M phenotype was identified for GBS isolates resistant to erythromycin and sensitive to clindamycin with simultaneous lack of flattening of the growth inhibition zone.

The detection of *erm*B and *mef*A/E genes was carried out according to the procedure used by Sutcliffe et al. (1996) with specific primers (Genomed S.A.) described in detail in our previous paper (Brzychczy-Wloch et al., 2010).

#### Serotyping

Gene-encoding capsular polysaccharide Ia, Ib, II–VIII detection was performed by multiplex PCR with specific primers (Genomed S.A.) according to Poyart et al.’s (2007) procedure described in our previous publication (Brzychczy-Włoch et al., 2012). The amplification product was separated in 2.0% agarose gel (Prona) with 5 μl ethidium bromide (Bio-Rad). The electrophoretic images were processed using Quantity One (Bio-Rad) software and analyzed with a GelDoc 2000 device (Bio-Rad).

#### Alp Gene Detection

The detection of genes encoding Alp proteins, namely *bca*, *rib*, *epsilon* (also called *alp*1), *alp*2/3, and *alp*4, was carried out with five pairs of primers (Genomed S.A.) according to the procedure used by Creti et al. (2004) for the multiplex PCR reaction described in our previous paper (Brzychczy-Włoch et al., 2012). To distinguish between the *alp*2 gene and *alp*3 gene, as well as to confirm *alp*3 presence, an additional PCR reaction, with the reverse primer Alp3 (5′-TTT TGG TTC GTT GCT ATC CTT AAG C-3′), was performed (Gherardi et al., 2007).

#### Genotyping by Pulsed-Field Gel Electrophoresis

The procedure was carried out according to that used by Tynkkynen et al. (1999), slightly modified and described in detail in our previous paper (Brzychczy-Wloch et al., 2010). Bacterial DNA had been digested with *Sma*I enzyme (Thermo Fisher Scientific), and restricted fragments had been separated in a CHEF-DR III device (Bio-Rad). The genetic similarity between the isolates was calculated using Molecular Analyst software (Applied Maths). The parameters used for clustering were the Jaccard index and unweighted pair-group method with arithmetic mean (UPGMA), where tolerance was 3% and optimization was 0.5%. Genotype patterns were compared according to the guidelines of van Belkum et al. (2007). Isolates with ≥80% similarity in PFGE patterns were considered to be indistinguishable and were assigned to the same subtype. The cut-off value was determined on the basis of the number and position analysis of the bands.

#### Adherence Abilities to the HMVEC-Bd Cells

The HMVEC-Bd cell culture (human bladder microvascular endothelial cell line) was carried out strictly according to the cell supplier (Lonza Rockland, ME, United States) (Kaszuba-Zwoińska et al., 2014). The cell line was maintained in an EGM-2MV BulletKit medium (Lonza Rockland, ME, United States) and cells were passaged by placing 60–80% confluent cell culture in trypsin-ethylenediaminetetraacetic acid (EDTA) solution (Sigma-Aldrich, St. Louis, MI, United States) for 10–15 min. Afterward, cells were suspended in Dulbecco’s modified Eagle’s medium with GlutaMAX (Gibco) supplemented with 10% fetal bovine serum (Gibco) and centrifuged at 416 *g* for 10 min. Cells were cultured in 8-well plates (ibidi) at the initial density of 5 × 10^4^ cells/well at the temperature of 37°C in the atmosphere of 90% humidity and with 5% of carbon dioxide concentration. The experiments were performed on cells in the logarithmic phase (approx. 5 × 10^6^ cells/well) of growth under the condition of ≥98% viability assessed by trypan blue exclusion test. Subsequently, GBS at the initial concentration of 1 × 10^8^ CFU/ml was cultivated in TSB and Dulbecco’s modified Eagle’s medium in ratio of 1:1 (250 μl:250 μl) on cell line for 4 h under aerobic conditions and fixed with paraformaldehyde and Gram stained. The adherence was semi-quantitatively determined by an estimation of the numbers of GBS cells in 20 randomly selected fields of vision (Bodaszewska-Lubas et al., 2013). Several hundred adherence cells of the studied GBS strains corresponded with strong adherence ( + ⁣ + ⁣ + ), several dozen adherence cells corresponded with moderate adherence ( + ⁣ + ), several bacterial cells corresponded with weak adherence (+), lack of visible adhered GBS cells indicated no adherence (−).

Cell line adherence was also visualized using a scanning electron microscope (SEM) according to the procedure described in our previous work (Golda-Cepa et al., 2016).

#### Immunoreactive Protein Detection

The detection of immunoreactive proteins was carried out by immunoblotting for 10 selected GBS strains, which belonged to various serotypes (Ia, Ib, II–V), coded diverse Alp genes (*rib*, *alp*2, *epsilon*), demonstrated two macrolide resistance phenotypes (cMLS_B_, iMLS_B_), and belonged to different PFGE clusters (2, 3, 4, 5, 6, 12, 13, 29, 30). Procedures of protein preparation and analysis have been previously described (Dobrut et al., 2018). They included cultivation in brain–heart infusion broth (Biocorp) at 37°C for 24 h. Afterward, the bacterial pellet whose final concentration equaled A600 nm = 1.0 was suspended in Tris–HCl (Merck) with the addition of sodium dodecyl sulfate in concentrations of 5–12.5% (Sigma-Aldrich, St. Louis, MI, United States) or directly in the electrophoresis buffer according to a slightly modified Heilmann’s procedure (Heilmann et al., 1996). Samples were sonicated three times for 5 min and centrifuged for 1 min in PBS.

Proteins were precipitated by three volumes of cold 95% ethanol (Avantor Performance Materials) during an overnight incubation at 4°C. Next, proteins were centrifuged and dissolved in water. Their final concentration was measured with the bicinchoninic acid assay (BCA) assay (Smith et al., 1985).

Protein homogenate was separated using sodium dodecyl sulfate polyacrylamide gel electrophoresis (SDS-PAGE) in Pep-Cell apparatus (Model 491 Bio-Rad) and next transferred to the Immobilon-P membrane. The membranes were blocked by 1% solution of bovine serum albumin (BSA; Thermo Fisher Scientific) in PBS for 1 h. Afterward, membranes were washed three times in PBS-T [PBS containing 0.25% Tween (Sigma-Aldrich, St. Louis, MI, United States)], and incubated with human serum samples isolated from GBS-positive [patients with GBS sepsis (GBS1, GBS2a, GBS2b), pregnant GBS carriers (PP4, PP6, PP7, PP8, PP9, SB3a, SB7, SB8), non-pregnant GBS carrier (NPP1)], and GBS-negative individuals [non-GBS carriers (SK1, SK2), and a patient with infection caused by *Streptococcus pyogenes* (GAS1) to exclude cross-reactivity] in dilution 1:300. Incubation was carried out at 37°C for 2 h. Unbound antibodies were washed three times with PBS-T, and next, membranes were incubated with secondary goat anti-human antibodies conjugated with alkaline phosphate (Sigma-Aldrich, St. Louis, MI, United States) in dilution 1:5,000 at room temperature for 1 h. After triple washing in PBS-T, the membranes were submerged for 5 s in a solution of nitroblue tetrazolium (NBT; Roth), 5-bromo-4-chloro-3-indolyl phosphate (BCIP; Roth), and MgCl_2_ (POCh) to visualize immunoreaction.

#### Statistical Analysis

The statistical analysis of the relationships between the examined parameters was carried out using the χ^2^ test, Fisher’s exact test, and Spearman’s rank correlation analysis in the IBM SPSS Statistics software. *P*-values of <0.05 were considered significant. The P-scores ranging from 0.05 to 0.1 were considered to be close to statistical significance (level of statistical tendency). The *V* value (Cramer’s V) determined an association between two nominal variables.

## Results

### Macrolide Resistance

**FIGURE 1:**
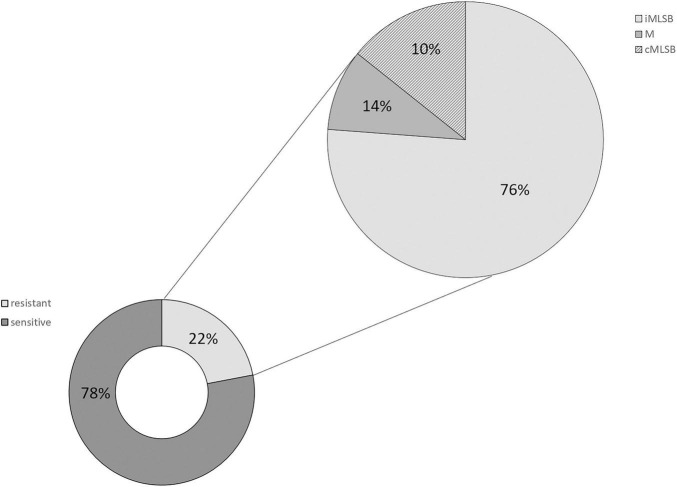
Percentage distribution of particular resistance phenotypes (cMLS_B_, iMLS_B_) (right side) of GBS isolated from UTI among the strains resistant to erythromycin and/or clindamycin (left side).

**FIGURE 2:**
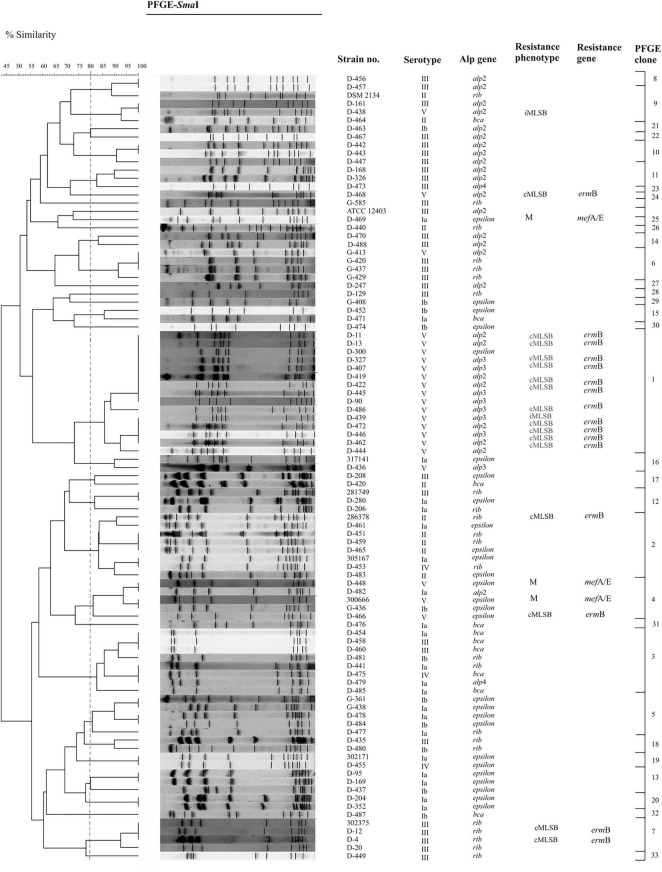
Dendrogram demonstrating the genetic relationship between 94 GBS isolates after restriction with *Sma*I enzyme. DNA fragments were separated by PFGE using the CHEF DR^®^II system. PFGE profiles were compared according to their percentage of similarity, estimated by the Jaccard coefficient and clustered by UPGMA using GelCompar II v.6.5 software. PFGE settings: optimization, 0.5%; tolerance, 3%. Strains belonging to the same group (PFGE clone) were clustered at a level of 80% similarity (dashed vertical line).

Among all examined GBS isolates, 21 (22%) were resistant to erythromycin, and/or to clindamycin (*n* = 17, 18%). Among the 21 strains demonstrating MLS_B_ phenotype, 76% showed cMLS_B_ phenotype, 14% belonged to M phenotype and 10% belonged to iMLS_B_ phenotype. One isolate was resistant to clindamycin and sensitive to erythromycin (Table 1 and Figures 1, 2). An analysis of the relationship between particular serotypes and resistance revealed a significant relation between serotype V and cMLS_B_ phenotype (*p* < 0.001, *V* = 0.40) (Table 1 and Figure 2). An examination of the relation between Alp genes and MLS_B_ phenotype showed significance between the frequency of the *alp*3 gene and cMLS_B_ phenotype (*p* = 0.004, *V* = 0.31) (Table 1 and Figure 2).

**TABLE 1:** Summary of molecular and phenotypic properties of clinical GBS strains isolated from patients with UTI.

GBS no.	Clinical number	Serotype	Alp gene	Resistance phenotype	*erm*B, *mef*A/E genes	PFGE clone
1	D-11	V	*alp2*	cMLS_B_	*erm*B	1
2	D-13	V	*alp2*	cMLS_B_	*erm*B	1
3	D-300	V	*epsilon*	–	–	1
4	D-327	V	*alp3*	cMLS_B_	*erm*B	1
5	D-407	V	*alp3*	cMLS_B_	*erm*B	1
6	D-419	V	*alp2*	–	–	1
7	D-422	V	*alp2*	cMLS_B_	*erm*B	1
8	D-445	V	*alp3*	cMLS_B_	*erm*B	1
9	D-90	V	*alp3*	–	–	1
10	D-486	V	*alp3*	cMLS_B_	*erm*B	1
11	D-439	V	*alp3*	iMLS_B_	–	1
12	D-472	V	*alp2*	cMLS_B_	*erm*B	1
13	D-446	V	*alp3*	cMLS_B_	*erm*B	1
14	D-462	V	*alp2*	cMLS_B_	*erm*B	1
15	D-444	V	*alp2*	cMLS_B_	*erm*B	1
16	286378	II	*rib*	cMLS_B_	*erm*B	2
17	D-461	Ia	*epsilon*	–	–	2
18	D-451	II	*rib*	–	–	2
19	D-459	II	*rib*	–	–	2
20	D-465	II	*rib*	–	–	2
21	305167	Ia	*epsilon*	–	–	2
22	D-453	IV	*epsilon*	–	–	2
23	D-483	II	*rib*	–	–	2
24	D-454	Ib	*bca*	–	–	3
25	D-458	V	*bca*	–	–	3
26	D-460	V	*bca*	–	–	3
27	D-481	V	*rib*	–	–	3
28	D-441	III	*rib*	–	–	3
29	D-475	Ib	*bca*	–	–	3
30	D-479	Ib	*alp4*	–	–	3
31	D-485	Ib	*bca*	–	–	3
32	D-448	V	*epsilon*	M	*mef*A/E	4
33	D-482	Ia	*epsilon*	–	–	4
34	300666	V	*alp2*	M	*mef*A/E	4
35	G-436	Ib	*epsilon*	–	–	4
36	D-466	V	*epsilon*	cMLS_B_	*erm*B	4
37	G-361	IV	*epsilon*	–	–	5
38	G-438	Ia	*epsilon*	–	–	5
39	D-478	IV	*epsilon*	–	–	5
40	D-484	Ia	*epsilon*	–	–	5
41	D-477	III	*rib*	–	–	5
42	G-413	V	*alp2*	–	–	6
43	G-420	III	*rib*	–	–	6
44	G-437	III	*rib*	–	–	6
45	G-429	III	*rib*	–	–	6
46	302375	III	*rib*	–	–	7
47	D-12	III	*rib*	cMLS_B_	*erm*B	7
48	D-4	III	*rib*	cMLS_B_	*erm*B	7
49	D-20	III	*rib*	–	–	7
50	D-456	III	*alp2*	–	–	8
51	D-457	III	*alp2*	–	–	8
52	D-161	III	*alp2*	–	–	9
53	D-438	V	*alp2*	iMLS_B_	–	9
54	D-464	II	*bca*	–	–	9
55	D-442	III	*alp2*	–	–	10
56	D-443	III	*alp2*	–	–	10
57	D-447	III	*alp2*	–	–	10
58	D-168	III	*alp2*	–	–	11
59	D-326	III	*alp2*	–	–	11
60	D-473	III	*alp4*	–	–	11
61	281749	III	*rib*	–	–	12
62	D-280	Ia	*epsilon*	–	–	12
63	D-206	Ia	*epsilon*	–	–	12
64	D-95	Ia	*epsilon*	–	–	13
65	D-169	Ia	*epsilon*	–	–	13
66	D-437	Ib	*epsilon*	–	–	13
67	D-470	III	*alp2*	–	–	14
68	D-488	III	*alp2*	–	–	14
69	D-452	Ib	*epsilon*	–	–	15
70	D-471	Ia	*bca*	–	–	15
71	317141	Ia	*epsilon*	–	–	16
72	D-436	V	*alp3*	–	–	16
73	D-208	III	*epsilon*	–	–	17
74	D-420	II	*bca*	–	–	17
75	D-435	III	*rib*	–	–	18
76	D-480	Ib	*rib*	–	–	18
77	302171	Ia	*epsilon*	–	–	19
78	D-455	IV	*epsilon*	–	–	19
79	D-204	Ia	*epsilon*	–	–	20
80	D-352	Ia	*epsilon*	–	–	20
81	D-463	Ib	*alp2*	–	–	21
82	D-467	III	*alp2*	–	–	22
83	D-468	V	*alp2*	cMLS_B_	*erm*B	23
84	G-585	III	*rib*	–	–	24
85	D-469	Ia	*epsilon*	M	*mef*A/E	25
86	D-440	II	*rib*	–	–	26
87	D-247	III	*alp2*	–	–	27
88	D-129	III	*rib*	–	–	28
89	G-408	Ib	*epsilon*	–	–	29
90	D-474	Ib	*epsilon*	–	–	30
91	D-476	Ia	*bca*	–	–	31
92	D-487	Ib	*bca*	–	–	32
93	D-449	III	*rib*	–	–	33
94	D-207	III	*alp2*	–	–	n/a

*PFGE, pulsed-field gel electrophoresis.*

The molecular detection of the *erm*B gene revealed its presence in every strain with cMLS_B_ phenotype, whereas the *mef*A/E gene had been identified in three GBS strains with M.

### Serotyping and Alp Gene Detection

An analysis of distribution of particular serotypes among all of the GBS strains showed that the most common serotype was serotype III (31%), followed by serotypes V (27%), Ia (17%), Ib (13%), II (8%), and IV (4%). Serotypes VI–VIII were not identified.

**FIGURE 3:**
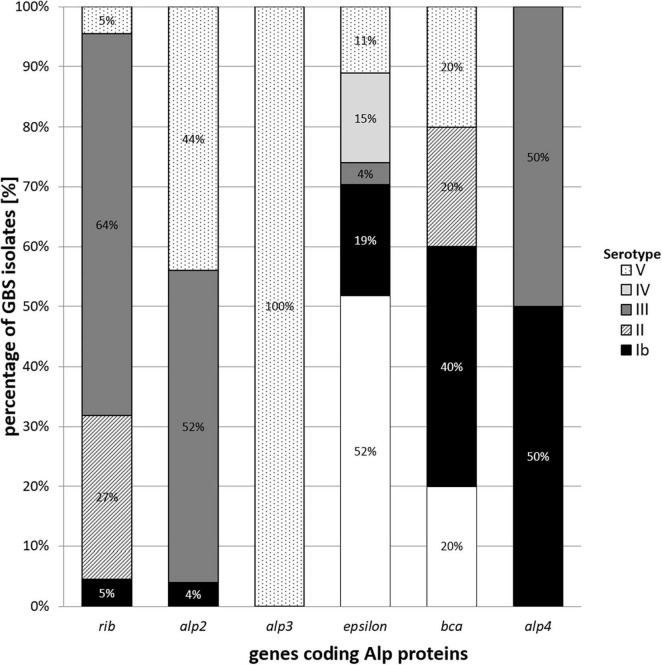
Percentage co-occurrence of various genes coding Alp proteins among Ia, Ib-V serotypes in group B streptococci isolated from UTI.

An examination of distribution of genes encoding Alp proteins showed the dominance of the *epsilon* gene (29%). The *alp*2 gene was common for 27% of GBS isolates, *rib* gene for 23%, *bca* gene for 11%, and *alp*3 gene for 8%, whereas *alp*4 was identified in 2% of the pool. Additionally, an analysis of co-occurrence of particular serotypes and Alp genes had been done. The most common co-occurrent types were serotype III and *rib* gene, serotype Ia and *epsilon* gene, serotype III and *alp*2 gene, serotype V and *alp*3 gene, and serotype V and *alp*2 gene (*p* < 0.001, *V* = 0.50). Detailed results have been presented in Table 1 and Figure 3.

### Pulsed-Field Gel Electrophoresis and Cluster Analysis

The PFGE method allowed to demonstrate high genetic diversity among the GBS isolates examined. At a cut-off level of 80%, 34 unique profiles were identified within all strains, among which 20 clusters included strain numbers ranging between 2 and 15 isolates and 14 demonstrated unique patterns (Table 1 and Figure 2). The cluster comparison showed that 15-multiple-strain (cluster no. 1) was most homogeneous; all isolates belonged to serotype V, exhibited cMLS_B_ phenotype in 93%, and encoded the following alp genes: *alp*2 (47%), *alp*3 (47%), and *epsilon* (6%). In cluster 2, 62% of the isolates belonged to serotype II, 25% demonstrated serotype Ia, and one strain belonged to serotype IV. A total of 62.5% encoded *rib* gene and 37.5% *epsilon*. Yet, cluster number 3 was dominated by serotype Ib (50%), followed by serotype V (37.5%), serotype III (12.5%), and the *bca* gene (50%). The less common ones were *rib* (25%), *epsilon*, and *alp*4 (12.5% each). The analysis of distribution of particular serotypes within the clusters was described as the ratio of pulsotypes to isolates belonging to particular serotypes (number of pulsotype/number of isolates) and revealed the biggest heterogeneity in serotype Ib (9/12) and IV (3/4), followed by serotype Ia (11/16), serotype II (5/8), and serotype III (18/29). The most homogenous was serotype V (7/25). Among Alp proteins, the most heterogeneous were *alp*4 (2/2), *bca* (6/10), and *epsilon* (15/27), followed by *rib* (12/22), and *alp*2 (13/25). The most homogenous was the *alp*3 gene (2/8).

### Bacterial Adherence to Bladder Endothelial Cells

**FIGURE 4:**
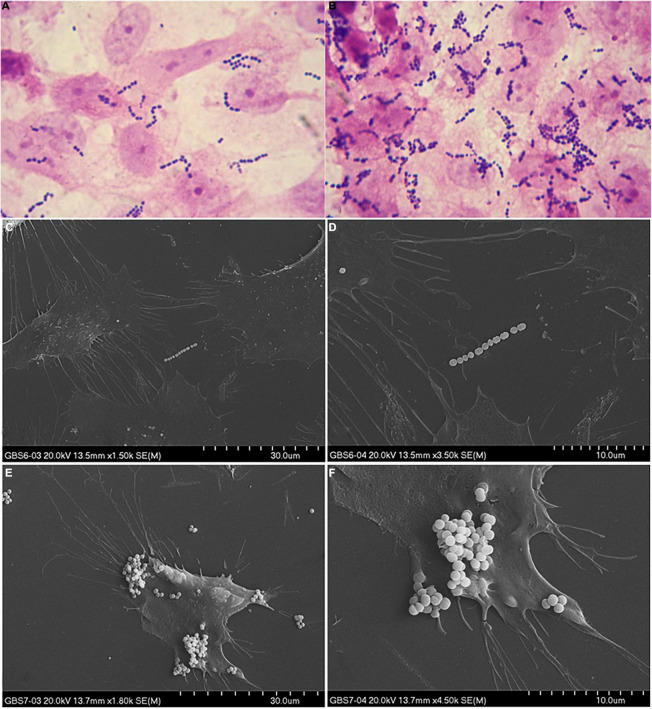
Example of weak **(A,C,D)** and moderate **(B,E,F)** adherence ability to the HMVEC cell line. Adherence ability in **(A,B)** was observed in optical microscope (1000×), whereas in **(C–F)** adherence was observed in SEM in 1800× (left side) and 4500× (right side) magnification.

**FIGURE 5:**
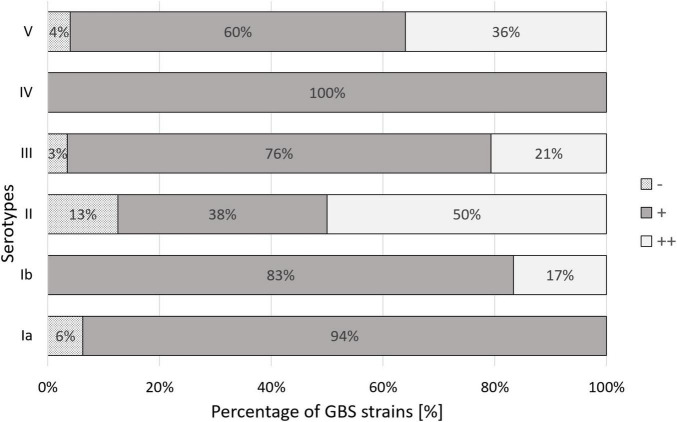
Percentage distribution of the degree of human bladder microvascular endothelial cell line (HMVEC-Bd) adherence among Ia, Ib-V serotypes of GBS. (–) – no adherence, (+) – weak adherence, (++) – moderate adherence. Significance of the association between serotype and bacterial adherence capacity equaled *p* = 0.044.

Adherence abilities were evaluated by estimation of the number of bacteria adhered to the HMVEC cell line. 74% of the investigated GBS isolates demonstrated weak (+) adherence capacity (Figures 4A,C,D), 22% adhered moderately ( + ⁣ + ) (Figures 4B,E,F), 4% did not adhere at all (−). No strain demonstrated strong adherence ability ( + ⁣ + ⁣ + ). Association between serotypes and bacterial cell adherence capacity was significant (*p* = 0.044, *V* = 0.28) (Figure 5) in opposition to the relationship between Alp genes and adherence capacity, where no significance was noticed.

### Immunoreactive Protein Detection

**FIGURE 6:**
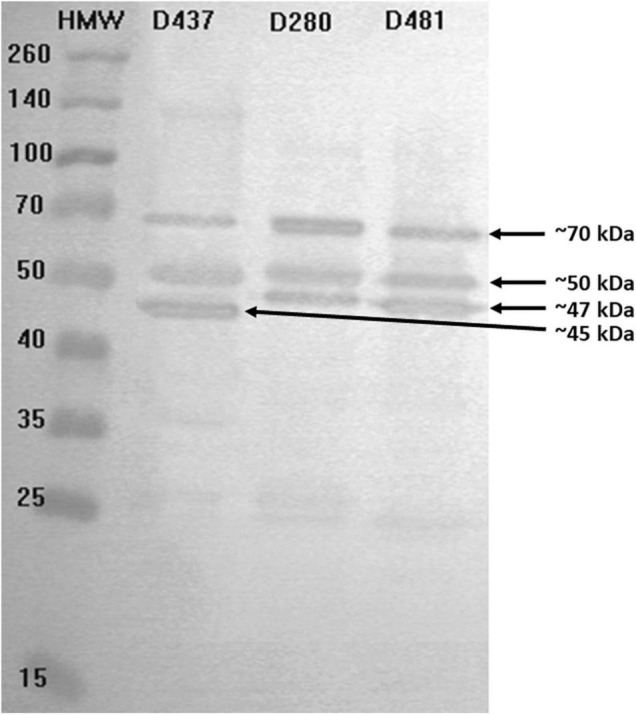
Exemplary results for immunoblotting of proteins from chosen GBS strains (strains no. D437, D280, D481) originating from UTI with visible protein bands by molecular weight 45, 47, 50, and 70 kDa. Immunoreactivity was studied in the presence of serum sample no. PP9 isolated from a carrier.

Immunoreactive GBS proteins were present in all studied strains (*n* = 10), which were genetically diverse. We observed that the most common proteins have molecular masses ranging from 45 to 47, 50, and 70 kDa (Figure 6); additionally, for some serum samples (GBS2a, PP4, PP7, SB3a, SB7), the presence of bands with masses approx. 25, 100, and 120 kDa was observed. What is worth noting is that even though immunoreactivity for GBS-positive serum was noticed, barely any reactivity was observed for GBS-negative control samples. In turn, for the GAS1 serum sample, which was isolated from a patient with an ongoing infection caused by *S. pyogenes*, no bands of the size 45 or 47 kDa were present, which may indicate high species specificity of these proteins. Interestingly, anti-GAS serum reacted with protein with mass approx. 120 kDa, which was undetectable by almost all other serums. Most of the studied strains revealed two-to-three immune-reactive proteins with GBS-positive serum, both from infection and carriage, except one (serum no. PP7), which detected seven immune-reactive proteins with molecular masses ranging from approx. 20 to approx. 140 kDa. No correlation between serotypes or other molecular factors, such as Alp genes or resistance phenotype or immune-reactive proteins pattern, was noticed. No regularity in immunoreactivity of proteins was noticed for serum from carriage and infection. For example, protein with molecular mass about 100 kDa was detected by serum no. GBS2a, which originates from ongoing GBS infection and by three sera from GBS carriage (sample nos. NPP1, PP7, and SB7).

## Discussion

GBS is one of the pathogens causing UTI. It can bring about severe issues in adults, especially elderly patients, in whom they can often take chronic form, which affects the excessive antibiotic intake. On the other hand, a widespread application of antibiotics, including erythromycin and clindamycin, results in growing resistance, which, in turn, reduces the effectiveness of treatment (Heelan et al., 2004; Rajagopal, 2009). Therefore, comprehensive characteristics of resistance profiles, virulence factors, such as capsular polysaccharide, or Alp proteins’ potential can provide meaningful data, which enrich the knowledge on GBS virulence and thus in the future may help to diagnose and treat infections caused by GBS and, ultimately, play an integral role in the development of alternative methods of infection treatment.

In recent years, an escalating therapeutic problem in GBS infection treatment has been the increasing resistance of these bacteria to macrolide antibiotics. In the population of Western Europe, the percentage of strains with the MLS_B_ phenotype, i.e., resistant to macrolides, lincosamides, and streptogramins B, ranges from 11.5 to 32% (Decoster et al., 2005; Phares et al., 2008; Murayama et al., 2009; Martins et al., 2011). This tendency was confirmed in our studies as, out of the pool of all clinical isolates of GBS, 22% demonstrated the MLS_B_ phenotype, of which the dominant type was cMLS_B_. The research group in Lindahl et al. (2005), when analyzing a pool of almost 5,000 clinical isolates of GBS in the United States, found resistance to erythromycin in up to 32% of isolates, while our investigation demonstrated resistance to this antibiotic in 22% of the strains; in turn, the incidence of clindamycin-resistant strains was similar and only varied by 3% (von Both et al., 2003). A slightly lower proportion of erythromycin resistance was reported in the Spanish population, where out of 212 patients, resistant strains were isolated from 30 people (Martins et al., 2011). However, Decoster et al. (2005) by studying over twice as many GBS isolates from Belgian pregnant women, presented a slightly lower percentage of isolates resistant to both erythromycin (16.7%) and clindamycin (11%), but the ratio of the cMLS_B_ and iMLS_B_ resistance phenotypes was almost the same and amounted to, analogously, 3.17 for the Belgian population and 3.15 for the Polish one. To the best of our knowledge, there are no data for the incidence of erythromycin-resistant strains isolated from Polish patients with UTI. However, when the results of this work are compared to the data presented in our previous publication, which characterized strains isolated from pregnant patients from the same region of Poland, an all-in higher proportion of strains resistant to erythromycin and clindamycin was observed in patients with UTI. There was also a greater incidence of isolates with UTI with the cMLS_B_ phenotype in the pool of resistant strains (Brzychczy-Wloch et al., 2010). In the paper, the authors only focused on investigating the resistance to erythromycin and clindamycin in order to determine the macrolide resistance phenotype. Monitoring the expansion of GBS phenotypes resistant to macrolides and clindamycin and the probable increase in this phenomenon indicates the need for regular assessment of the susceptibility of all isolates to these antibiotics. Regular epidemiological monitoring can be of great importance in predicting future phenotypic changes in group B streptococci.

The distribution of individual serotypes varies and depends on many factors such as latitude or type of infection. According to literature data, serotype III is predominant in UTIs (Gherardi et al., 2007; Sullivan et al., 2017). The same result was obtained in our research, in which serotype III was identified in 31% of the strains, while serotype V, considered as the most virulent, was the second most often isolated serotype and it was present in 27% of the GBS isolates. Third, the most commonly occurring serotype was serotype Ia, which appeared in 17% of the GBS isolates, and that corresponded with the results obtained by Gherardi et al. (2007), who noticed the same amounts among a comparable number of the strains isolated from various infection types, among others, from UTI in Italy.

Analysis of distribution of particular serotypes in the group of resistant GBS strains indicated the significant predominance of serotype V over others (*p* < 0.001 for cMLS_B_). This tendency was also demonstrated by von Both et al. (2003), who studied a German population of both pregnant women and neonates, despite the fact that the proportion was twofold higher in the Polish population. Yet, in a group of patients hospitalized in South Korea, serotype V was identified in 43.6% of resistant strains, while our study pointed to an amount as high as 80% (Uh et al., 2004). Furthermore, the evaluation of relationships between the MLS_B_ resistance phenotype and the incidence of genes encoding Alp proteins displayed a significant relation of the *alp*3 gene with the cMLS_B_ phenotype (*p* = 0.004). A similar result was obtained by Gherardi et al. (2007). The data presented in this study also corresponded to the observations from our previous investigation in which serotype V and alp protein were predominant in a pool of cMLS_B_ strains isolated from pregnant women (Brzychczy-Włoch et al., 2012).

Proteins present on the GBS cell surface play an important role at various stages of infection (Kong et al., 2002; Creti et al., 2004), and the co-occurrence of particular serotypes and specific protein profiles is evolutionarily conditioned. It is hypothesized that certain combinations of proteins and polysaccharides are preferred by the immune system because they are a better match, and it is also suggested that individual polysaccharides can protect surface proteins against antibodies (Lindahl et al., 2005). In our studies, we have demonstrated a significant co-occurrence of serotype V and *alp*2 gene, serotype III and *rib* protein, as well as serotype Ia and *epsilon*, which is confirmed by other research works. A research team headed by Piccinelli, which based its study on a similar number of GBS clinical isolates collected from Italian patients with UTI, recorded an overwhelming prevalence of the same three combinations (Piccinelli et al., 2015). Gherardi et al. (2007), while investigating clinical isolates originating from various infection types, i.e., UTI, recorded a similar frequency of co-occurrence of serotype Ia and *epsilon* protein of about 50% and a high proportion of strains with serotype III and the *rib* gene (60.1%). In our study population, 44% of isolates represented this pattern. Despite the fact that there is a lack of data concerning the frequency of co-occurrence of particular serotypes and proteins in UTI in the Polish population, our previous studies, which were carried out on a group of pregnant women, demonstrated that the most often isolated GBS strains represented serotype Ia and *epsilon* and serotype III and gene *rib* (Brzychczy-Włoch et al., 2012).

Bacterial adherence to host cells plays an important role in colonization and pathogenesis. Therefore, a comprehensive description of this phenomenon under *in vitro* conditions may provide valuable information on the mechanism of infection caused by GBS. As can be seen from our observations, despite the fact that the general adherence of GBS strains to HMVEC cell line surface was weak, the abilities of isolates belonging to serotype II were significantly higher. However, it does not correspond to the results achieved by other researchers. Bodaszewska-Lubas et al. (2013), when conducting studies on two cell lines, human colon adenocarcinoma cell line (HT-29) and the human epidermoid vulvo-vaginal cells (A-431), showed the dominance of serotypes Ia and III among strains isolated from pregnant women and newborns. Furthermore, Botta (1979) revealed the dominance of serotype III in the adherence to epithelial cells of the human vagina. These discrepancies may result from the use of different types of cell lines in each of the experiments. Additionally, we did not carry out the bacterial growth curve. We assumed that regardless of the GBS serotype observed in our previous studies, no differences in inoculum after 18–24 h of incubation can be translated into the experiments described in this article. However, as shown by Malin and Paoletti (2001), the growth rate impacts the bacterial adherence. Therefore, it seems to be warranted to include the bacterial growth curve in the further studies.

Detection of immunoreactive GBS proteins representative of strains isolated from adult patients with UTI demonstrated the presence of several immunoreactive proteins whose molecular masses ranged from approx. 20 to 140 kDa, whereas two of them—with masses approx. 45 and 47 kDa were the most common for the GBS-positive sample, and what is worth noting is that they were not detectable by serum from the patient with the ongoing infection caused by *S. pyogenes.* This is in opposition to protein with mass approx. 50 kDa, which was also present in the GAS sample. Thus, it may indicate not only immunoreactivity but also their species specificity. To the best of our knowledge, it is the first study to indicate immunoreactive GBS proteins for UTI in adults.

In our previous paper, we detected immunoreactive GBS proteins on an extended number of samples of isolates (*n* = 60) (Brzychczy-Wloch et al., 2013). Selected strains originated from both newborns and adults (pregnant and not pregnant), various infection sources, including UTIs in newborns, from different regions of Poland, and they were genetically diverse—they demonstrated various serotypes, Alp genes, sequence types, and macrolide resistance phenotypes. The immunoreactivity of the isolated proteins was studied in the presence of serum samples isolated from GBS-positive sera, both from infections and carriage, and GBS-negative sera, which constituted control, and moreover, they originated from vascular and umbilical cord blood. However, despite the number of various factors, some proteins were common for all of the studied strains. Interestingly, these highly immunoreactive and specific proteins represented molecular masses of about 45–50 kDa; therefore, they covered our observations described in this article. Additionally, these proteins had been subjected to sequencing, and next, highly specific epitopes were characterized (Dobrut et al., 2018; Pyclik et al., 2018). The amino acid sequencing of chosen bands of interest carried out by Brzychczy-Wloch et al. (2013) allowed to identify the following proteins: aldehyde dehydrogenase (50.6 kDa), enolase (47.4 kDa), trigger factor (47 kDa), and elongation factor Tu (44 kDa). Therefore, we can hypothesize that our proteins with masses 45 and 47 kDa can be the same proteins. Thus, it may indicate that the proteins with masses approx. 45 and 47 kDa, of which immunoreactivity and selectivity had also been confirmed in this study, demonstrate the features, allowing us to consider them as potential biomarkers in immunodiagnostic assay, in which protein antigens constitute the detective molecules. This is not only, as was shown by Dobrut et al. (2018) and Pyclik et al. (2018), is GBS carriage in pregnant women but also in UTI diagnosis. Nevertheless, it is necessary to underline that further examination such as identification of detected immunoreactive proteins as well as investigation carried out on extended number of samples is required.

## Conclusion

Undoubtedly, UTIs constitute a severe clinical problem. In spite of the fact that *S. agalactiae* is not the main pathogen behind UTI, a comprehensive knowledge of its virulence factors and resistance mechanisms may provide data that in the future can significantly contribute to enhancing the therapy for infections caused by this pathogen through appropriate selection of an effective antibiotic therapy. The *S. agalactiae* clinical isolates we examined were characterized by a high degree of resistance to antibiotics from the classes of macrolides, lincosamides, and streptogramins B, as well as high genetic diversity. Owing to the fact that, until now, similar studies in the Polish and the Central European population in general have not been carried out, these findings can provide valuable information and expand the knowledge of the contribution of different GBS serotypes to UTI infections in adults. In turn, the detection of immunoreactive and species-specific GBS proteins can deepen our knowledge in the field of GBS epidemiology, and in the future, they may be considered as potential biomarkers in UTI diagnosis as well as components of a subunit vaccine directed against GBS infection. What is worth underlining is, despite the variability among GBS strains, according to the comparable molecular masses, with high probability, the same proteins dominate both in UTI and in GBS sepsis and GBS carriage. Nevertheless, more data including protein identification and extension of sample number are undoubtedly required.

## Data Availability Statement

The raw data supporting the conclusions of this article will be made available by the authors, without undue reservation.

## Ethics Statement

The studies involving human participants were reviewed and approved by the Jagiellonian University Bioethical Committee decision No. KBET/153/B/2014. The patients/participants provided their written informed consent to participate in this study.

## Author Contributions

AD conducted DNA isolation, molecular detection of resistance genes, serotyping, detection of Alp genes, performed the PFGE procedure, and drafted the manuscript. DO collected samples and analyzed PFGE results. EB and SG carried out bacterial proteins isolation and conducted detection of the immunoreactive proteins. JK-Z carried out cell line adherence studies. MG-C carried out SEM analyses. AG was a supervisor and helped to draft the manuscript. MB-W designed the concept of the study, obtained funds for research, coordinated the project, analyzed the results, and coordinated and helped to draft the manuscript. All authors read and approved the final manuscript.

## Conflict of Interest

The authors declare that the research was conducted in the absence of any commercial or financial relationships that could be construed as a potential conflict of interest.

## Publisher’s Note

All claims expressed in this article are solely those of the authors and do not necessarily represent those of their affiliated organizations, or those of the publisher, the editors and the reviewers. Any product that may be evaluated in this article, or claim that may be made by its manufacturer, is not guaranteed or endorsed by the publisher.

## Footnotes

1
www.eucast.org
